# Ultra-fast pulse propagation in nonlinear graphene/silicon ridge waveguide

**DOI:** 10.1038/srep16734

**Published:** 2015-11-18

**Authors:** Ken Liu, Jian Fa Zhang, Wei Xu, Zhi Hong Zhu, Chu Cai Guo, Xiu Jian Li, Shi Qiao Qin

**Affiliations:** 1 College of Optoelectronic Science and Engineering, National University of Defense Technology, Changsha, Hunan 410073, China; 2 State key laboratory of High Performance Computing, National University of Defense Technology, Changsha, Hunan 410073, China; 3 College of Science, National University of Defense Technology, Changsha, Hunan 410073, China

## Abstract

We report the femtosecond laser propagation in a hybrid graphene/silicon ridge waveguide with demonstration of the ultra-large Kerr coefficient of graphene. We also fabricated a slot-like graphene/silicon ridge waveguide which can enhance its effective Kerr coefficient 1.5 times compared with the graphene/silicon ridge waveguide. Both transverse-electric-like (TE-like) mode and transverse-magnetic-like (TM-like) mode are experimentally measured and numerically analyzed. The results show nonlinearity dependence on mode polarization not in graphene/silicon ridge waveguide but in slot-like graphene/silicon ridge waveguide. Great spectral broadening was observed due to self-phase modulation (SPM) after propagation in the hybrid waveguide with length of 2 mm. Power dependence property of the slot-like hybrid waveguide is also measured and numerically analyzed. The results also confirm the effective Kerr coefficient estimation of the hybrid structures. Spectral blue shift of the output pulse was observed in the slot-like graphene/silicon ridge waveguide. One possible explanation is that the blue shift was caused by the ultra-fast free carrier effect with the optical absorption of the doped graphene. This interesting effect can be used for soliton compression in femtosecond region. We also discussed the broadband anomalous dispersion of the Kerr coefficient of graphene.

Nonlinear effects in CMOS-compatible integrated optical devices are of great significance as they can be explored to realize a variety of functionalities ranging from all-optical data processing to light generation. The optical nonlinearity of most materials is generally weak. For example, silica in fiber has a Kerr coefficient n_2_ in the order of 10^−20^ m^2^/W in the communication band^1^ and silicon used in CMOS integrated devices has a Kerr coefficient n_2_ in the order 10^−18^ m^2^/W, which is about two orders larger than that of silica^2^,^3^. As a result, lots of efforts have been made to enhance the nonlinear effects. Enhancement of nonlinear effects, such as optical THG in silicon waveguides, can be achieved by the mechanism of slow-light^4^,^5^,^6^ since decrease of group velocity could cause increase of light intensity in the waveguide. However, for a static photonic structure, decrease of the group velocity would increase extrinsic propagation loss^7^ and narrow the bandwidth which depends on the number of different resonant modes^8^. Enhancing nonlinear effects by resonant structures faces similar problem. Another way to achieve enhancement of light-matter interactions is the use of metallic structures^9^,^10^ where light localization and field enhancement can be realized due to the excitation of surface plasmons. However, it unavoidably causes an increase of ohmic losses.

Recently, graphene has attracted enormous attention for photonic and optoelectronic applications. Besides its many other novel optical properties, graphene has broadband nonlinear optical response^10^,^11^,^12^ and a huge n_2_ which is in the order of 10^−13^ m^2^/W at the wavelength 1.0 μm^13^. Moreover, graphene is CMOS compatible and can be integrated with different optical devices including waveguides and fibers. The feasibility of easy integration of graphene with CMOS integrated optical devices^14^,^15^,^16^,^17^,^18^ provides a new way of nonlinear enhancement in CMOS-compatible integrated devices. Theoretical results also show that ultra large nonlinear parameter can be achieved in hybrid graphene/silicon waveguides^19^. In this paper, we report the femtosecond laser propagation in a hybrid graphene/silicon ridge waveguide and demonstrate the ultra-large n_2_ of graphene without employing slow light or resonant structures.

## Results

**Figure 1:**
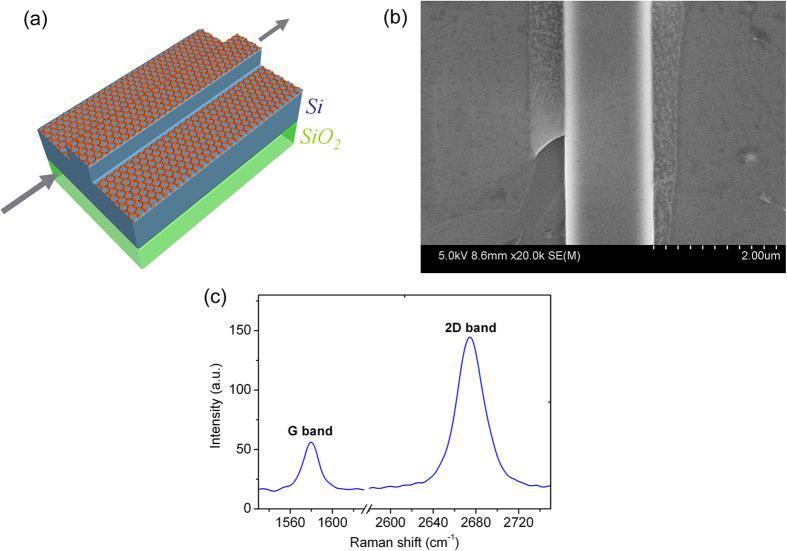
(**a**) Schematic of ultra-fast pulse propagation along the hybrid graphene/silicon ridge waveguide. (**b**) SEM image of the graphene silicon ridge waveguide. (**c**) Raman spectra of the graphene sample on SOI.

Schematic of the femtosecond laser propagation in the hybrid waveguide is shown in Figure 1a. Monolayer graphene is transferred on top of silicon ridge waveguide to form hybrid graphene/silicon ridge waveguide. The complex refractive index 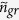 of intrinsic graphene in the communication wavelength range can be obtained from^20^


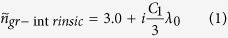


Where *C*_1_ ≈ 5.4 μm^−1^ and 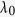 is wavelength in vacuum. At 1.56 μm, the complex refractive index of graphene is about 3.0 + 2.8 i which corresponds to a high absorption of light. The absorption of graphene can be controlled by tuning its Fermi level through chemical doping^14^ or electrical doping^15^. For ideal N-doped graphene with the Fermi level above 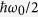 or P-doped graphene with the Fermi level below 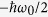, where 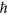 is the Plank constant divided by 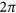 and 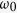 is the angular frequency of light, there should be no absorption of graphene for the photons with energy 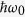. However, although photon absorption is reduced due to Paul blocking after doping, the absorption usually gets reduced only by about one order since defects in graphene is inevitable. In this paper we assume that the refractive index of the chemically N-type doped graphene is:


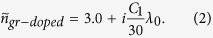


**Figure 2:**
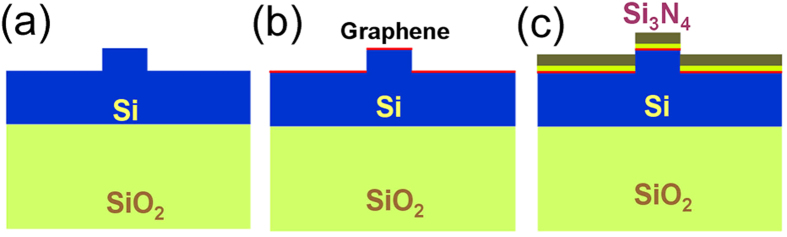
Schematic of a silicon ridge waveguide, a graphene/silicon ridge waveguide and a graphene/silicon slot-like ridge waveguide. (**a**) A silicon ridge waveguide with a width of 1.5 μm and an etching thickness of 0.4 μm. (**b**) A monolayer graphene is layered on the silicon ridge waveguide shown in (**a**). (**c**) After transfer of graphene to the ridge waveguide, a silica layer with a thickness of 40 nm and a Si_3_N_4_ layer with a thickness of 200 nm are deposited on the graphene layer.

**Figure 3:**
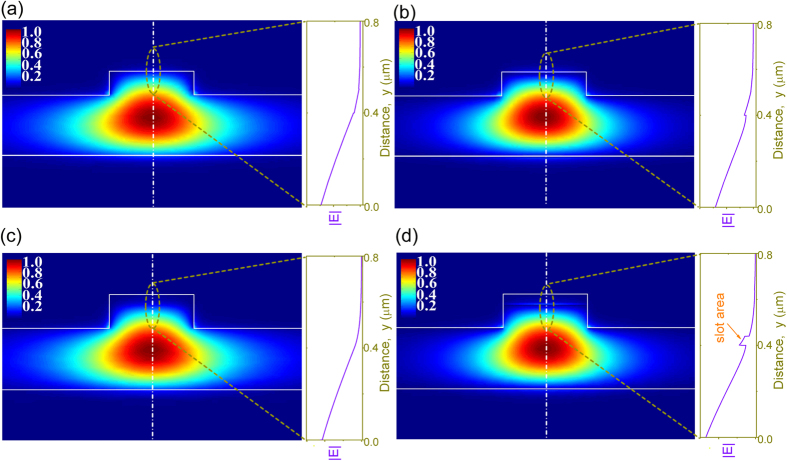
Normalized field distributions of TE-like and TM-like modes. (**a**) TE-like mode and (**b**) TM-like mode distributions of the hybrid graphene/silicon ridge waveguide as shown in Figure 2b. (**c**) TE-like mode and (**d**) TM-like mode distributions of the hybrid graphene/silicon slot-like ridge waveguide as shown in Figure 2c. In the right side of each figure, it shows the electrical field profile along the white dashed lines in the dashed dark yellow circle.

A 1.5 μm thick SOI wafer is used to fabricate the ridge waveguides with width of 1.5 μm and thickness about 0.4 μm (Figure 2a). Graphene is layered on the surface of the silicon ridge waveguide (Figs 1b and 2b). The single-layer graphene can be clearly identified by the Raman spectra (Figure 1c). Numerical simulations show that the complex refractive indices of the ridge graphene/silicon waveguide are 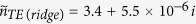 for TE-like mode (Figure 3a) and 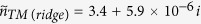 for TM-like mode (Figure 3b). The propagation distance is about 20 mm, thus we can neglect the propagation loss for a waveguide with a length of 2 mm.

The nonlinear parameters of graphene is ultra large. The third order nonlinear susceptibility 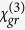 of graphene can be obtained from^13^,^19^ 
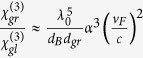
, where 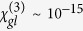 esu, which is third order nonlinear susceptibility of glasses, *d*_*B*_ is the Bohr radius, *d*_*gr*_ is thickness of graphene, *α* is the fine structure constant, and 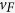 is the Fermi velocity. If *d*_*B*_ ~ 1 Å and *d*_*gr*_ ~ 3.4 Å, We estimate that 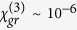 esu when 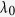 equals 1.56 μm. 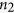 can be obtained from 
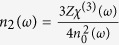
, where *Z* is the intrinsic impedance and *Z* = 377 Ω, 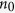 is the linear refractive index of the material. The corresponding Kerr coefficient is *n*_*2*(graphene)_ ~ 10^−12^ m^2^/W at 1.56 μm, that is 6 orders larger than that of silicon^2^ which is about *n*_*2*(silicon)_ ~ 4.0 × 10^−18^ m^2^/W. With a monolayer graphene cladding on the ridge waveguide (Figs 1b and 2b), the effective Kerr coefficient 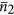 can be calculated from 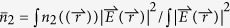 Where *n*_*2*_ is the Kerr coefficient of the material. We can find that electrical field |E| distributions of different modes in different structures can affect the value of *n*_*2*_. Figure 3a,b show the electrical field distributions of TE-like and TM-like modes of the silicon ridge waveguide (Figure 2b). The electrical field distributions of the two modes are similar. Most of the electrical fields are confined within the waveguide. With electrical field distributions shown in Figures 3a,b, we can obtain that the effective 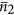 are about 1.3 × 10^−17^ m^2^/W for TE-like mode (Figure 3a) and 1.4 × 10^−17^ m^2^/W for TM-like mode (Figure 3b), respectively. This results show that with only one single layer graphene, the 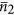 of the hybrid ridge waveguides are three times greater than that of silicon.

**Figure 4:**
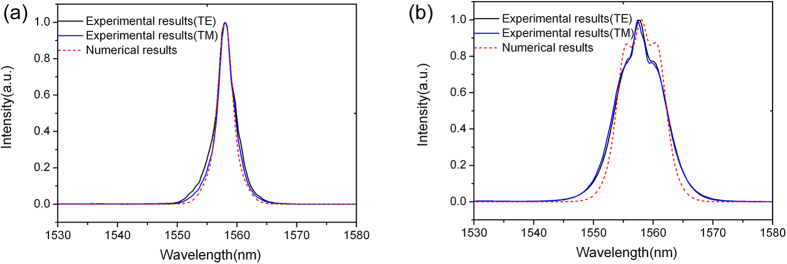
Experimentally measured and numerically calculated spectra of the output femtosecond pulses propagating along the silicon ridge waveguide as shown in Figure 2a. (**a**) The average output power is 0.5 mW. (**b**) The average output power is 2.0 mW.

In order to verify the 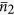 increase from the silicon ridge waveguide (Figure 2a) to hybrid graphene/silicon waveguide (Figure 2b), femtosecond laser pulses were coupled to the two types of waveguides respectively to observe the spectral evolution. The input pulse width is about 300 fs and the repetition rate is 60M Hz. We firstly coupled the pulse to the silicon ridge waveguide (Figure 2a) with a length of 2 mm. Figure 4a shows the spectral evolution with an average power of 0.5 mW (~8.3 pJ per pulse). The figure does not show obvious phase change at 0.5 mW. Then we increase the injected power 4 times higher to 2.0 mW, and SPM can be observed in Figure 4b. Both Figures 4a,b show that there is almost no polarization dependence on TE-like mode and TM-like mode. This is because that most of the electrical fields of both TE-like mode (Figure 3a) and TM-like mode (Figure 3b) are confined in the silicon ridge waveguide, thus, without covering of graphene, the two modes have nearly the same effective 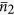, which is assumed to be 4.0 × 10^−18^ m^2^/W in the numerical simulation as shown in Figure 4.

**Figure 5:**
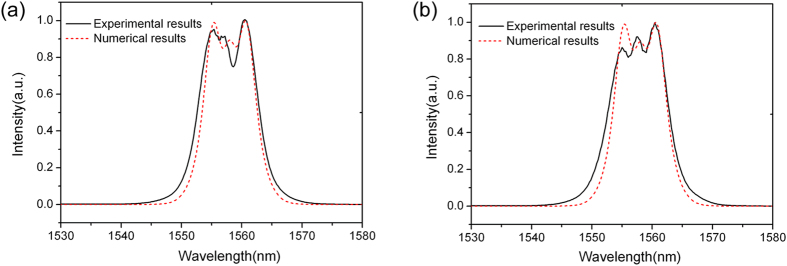
Femtosecond pulses propagating along the graphene/silicon ridge waveguide as shown in Figure 2b. The average output power is 0.50 mW. (**a**) TE-like mode propagation. (**b**) TM-like mode propagation.

After the silicon ridge waveguide measurement, we then transferred one single layer graphene to the same ridge waveguide to form graphene/silicon hybrid waveguide (Figure 2b). Figure 5 describes the results of TE-like mode and TM-like mode propagating along the hybrid waveguide. In the numerical simulations as shown in Figure 5, 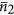 are assumed to be 1.3 × 10^−17^ m^2^/W and 1.4 × 10^−17^ m^2^/W respectively. The propagation length is 2 mm. The numerical simulations agree with the experimental results. Comparing Figure 4a with Figure 5, we can find that the pulses in the waveguides both have the same average power of 0.5 mW, while the latter shows obvious nonlinear effects for both TE-like and TM-like modes. In Figure 4b, the average pulse power (2.0 mW) is 4 times larger than that shown in Figure 5 (0.5 mW), however, the latter shows a stronger SPM spectral broadening effect. This indicates that the introduction of graphene indeed increases the Kerr coefficient of the waveguide. The maximum phase change 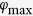 at the pulse center shown in Figures 5a,b is about 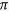 when comparing with the reference^1^. Here 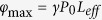, where 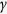 is nonlinear parameter, 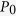 is the incident power, 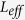 is the effective length.

From equation 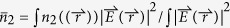, it can be found that the monolayer graphene position is the area with ultra-huge nonlinear parameters. Increase the electric field intensity at the graphene area can increase the 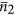 effectively. While in the structure shown in Figure 2b, the electrical fields at the surface of the waveguide are evanescent fields and are quite weak for both TE-like and TM-like modes (Figures 3a,b). Then the ultra-huge nonlinear optical properies of graphene are not utilized efficiently in this case.

Slot waveguide is a kind of waveguide that the electrical field is mainly located within the lower index material which is sandwiched between high index materials^19^,^21^. Thus we can use slot-like waveguide to enhance light density at the graphene layer. We fabricated a slot-like ridge waveguide by depositing 40 nm thick SiO_2_ and 200 nm Si_3_N_4_ on top of the graphene (Figure 2c). As shown in Figures 3c,d, there is a sharp increase of |E| at the silicon/graphene/silica interface for TM mode (Figure 3d, right side). This is because that the y component of the electrical displacement is continuous at the silicon/graphene/silica surface, thus the y component of the electrical field in the low index layer around the interface corresponds to higher amplitudes against that in the high index silicon layer. Numerical simulations show that the 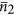 are about 1.5 × 10^−17^ m^2^/W for TE-like mode and 2.2 × 10^−17^ m^2^/W for TM-like mode which is about 5 times greater than that of silicon. To verify the increase of 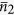, we also studied the spectra evolution of the ultra-fast pulse propagating through the waveguide.

**Figure 6:**
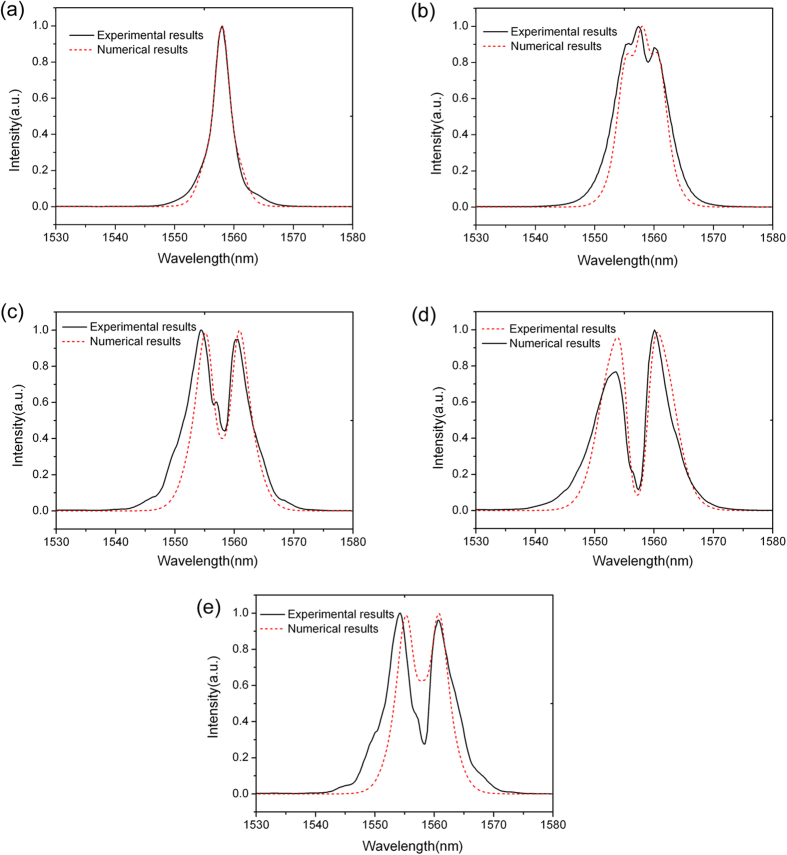
Femtosecond pulses propagating along the graphene/silicon slot-like ridge waveguide as shown in Figure 2c. (**a**–**d**) TM-like modes with average output power at 0.30 mW, 0.38 mW, 0.44 mW and 0.50 mW, respectively. (**e**) TE-like modes propagating along the same graphene/silicon slot-like ridge waveguide with output power 0.50 mW.

Figure 6 shows the pulse transmission spectra along the same waveguide (Figure 2b) but with SiO_2_ and Si_3_N_4_ deposition (Figure 2c). Figure 6a–d show the spectra with average pulse power of 0.30 mW (~5.0 pJ), 0.38 mW (~6.3 pJ), 0.44 mW (~7.3 pJ) and 0.50 mW (~8.3 pJ), respectively. In the simulation, the effective Kerr coefficients are 2.2 × 10^−17^ m^2^/W and kept invariant. In Figure 6a there is no obvious spectra blue shift when the peak pulse power is below 19 W, the measured results and the numerical simulations agree well with each other. These results (Figures 6a–d) also demonstrate the exactness of the effective 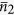 in our previous analysis.

Figure 6d,e show the transmission spectra of the TM-like mode and TE-like mode with average pulse power of 0.50 mW, respectively. The spectra are significantly broadened by SPM compared with that shown in Figure 4a. As shown in Figure 3d and discussed above, with the increase of light-graphene interaction for TM-like mode, Figures 6d,e show that the effective Kerr coefficients 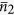 have strong polarization dependence on TE-like mode and TM-like mode. 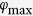 of TM-like mode shown in Figure 6d is about 1.5 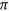. This indicates that the 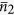 of the structure (Figure 2c) is increased about 1.5 times compared with the structure shown in Figure 2b, accompanied with spectral broadening.

Figure 6e shows that there is a little bit larger 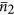 of the waveguide from the experimental result than the numerical simulations. We note that the graphene is layered not only on the top of the waveguide, but also partially on both sides of the waveguide (Figure 1b), thus the 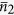 should be a little bit larger than the numerically calculated 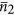 for TE-like mode.

The experimental results in Figures 6d,e exhibit blue shift. The blue shift in Figure 6d is about 1.0 nm at high frequency region, and output pulse in Figure 6d shows asymmetry shape. We know that free carrier effect, such as free carrier dispersion (FCD) and free carrier absorption could lead the output pulse to be asymmetry, especially FCD effect, it can lead to the refractive index decreasing and thus cause acceleration of the pulse^22^,^23^,^24^, and thus lead to the blue shift of the output pulse. FCD has been used to demonstrate soliton compression in silicon photonic crystal waveguides^23^,^24^. However, the free carrier response in silicon is not an instantaneous response, and picosecond pulses are needed to achieve this effect. Blue shift of output pulse caused by free-carrier-induced pulse acceleration was not observed with femtosecond pulses in silicon photonic crystal waveguides in recent studies^24^. In the experiment^24^, the light power is 40 W, group index is greater than 24, the mode area is 0.47 μm^2^, and the duration was 100 fs. While in the measurement shown in Figure 6d, the light power is 25 W, the group index is 3.4, the mode area is 1.0 μm^2^ and the duration is 300 fs. The parameters in Figure 6d is smaller than that in the previous experiment, except that the duration of the pulse is 3 time longer. We can not exclude free carrier effects in silicon in 300 fs duration. But the most possible explanation is that the blue shift effect was induced by the free carrier effect after the optical absorption of the doped graphene. As mentioned above, although the graphene was highly doped, the optical absorption was still inevitable. The reponse of free carriers in graphene is also non-instantaneous, but is quite quick. The hot carriers of graphene have a short lifetime ~150 fs and longer lifetime > 1 ps^25^. Thus free carriers in graphene can response the femtosecond pulse in our experiment and may cause the blue shift. However, the physics of ultra-short pulse propagation in graphene/silicon waveguide is much richer than in silicon waveguide and we need further study to obtain quantitative results.

## Discussions

During the pulse propagation, there should be other nonlinear effects such as two photon absorption, and group-velocity dispersion^1^. Such nonlinear effects are not considered in numerical simulations since they do not play important roles with a large mode area 1.0 μm^2^ of the ridge waveguide than that of the strip waveguide.

From Figure 3, it can be found that the electrical field intensity at the ridge waveguide surface is much weaker than that of other types of silicon waveguides^26^. However, with only one layer graphene, the hybrid structure shows great nonlinearity enhancement up to 5 times, this also demonstrates that the graphene itself has ultra-large nonlinear parameters. By depositing graphene and insulator layer alternatively, we can achieve multilayer gaphene structures to enhance the nonlinear coefficients of the hybrid waveguide. Since the graphene is less than 1 nm thick, the thickness of multilayer graphene structures can be controlled less than a few tens of nanometers. This also indicates that graphene is a potential nonlinear optical material for future on-chip applications.

We should note that in theoretical simulations, it is assumed that 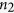 of graphene is fixed at communication wavelength 1558 nm. However, from the theoretical analysis, 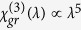, and thus 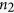 of graphene varies with at different wavelengths. This can be considered as material dispersion. It is not the linear part of the refractive index dispersion, but nonlinear refractive index dispersion, and actually, the 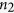 of graphene shows an anomalous dispersion. For a femtosecond pulse, the band in frequency region is very wide. The central wavelength of the input pulse is 1558 nm, we can estimate the ratio between 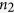 of graphene at 1565 nm and 1545 nm 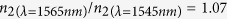. This significant change should also affect the dispersion during the spectral evolution. We think that the 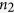 dispersion of graphene is a very interesting and useful effect that can be used in the future soliton generation in silicon based on-chip devices. The 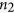 of graphene has a broadband anomalous dispersion region, and the 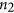 of graphene is ultra-huge. The anomalous dispersion of 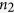 can counterbalance SPM induced dispersion of the pulse in silicon nanowire waveguides. This provides the possibility that we can design zero-dispersion materials without designing complicated structures such as dispersion engineering with photonic crystals.

## Conclusions

We have studied ultra-fast femtosecond pulse propagation in nonlinear graphene/silicon ridge waveguide, and obseved great spectral broadening due to SPM, and blue shift due to free carriers generated by the absorption of doped graphene. Although the electrical field at the surface of the ridge waveguide is evanescent fields and the pulses have quite weak interactions with graphene, the hybrid waveguides nonlinear coefficient was enhanced several times larger. This is a direct demonstration that graphene has an ultra-large nonlinearity and can be integrated with CMOS-compatible silicon devices. The results provide a new way for further on-chip nonlinear device design and applications such as soliton compression in silicon nanowire waveguide.

## Methods

### Definition of the waveguide’s electrical mode area

The definition of the effective electrical mode area 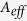 can be found from the reference^21^. In numerical simulations, the thickness of graphene is assumed to be 1 nm. The effective electrical mode areas of the ridge waveguide and the ridge-slab waveguide are estimated to be 1 μm^2^ obtained from numerical calculation.

### Definition of the propagation length of the waveguide

The light propagation length of the waveguide is obtained by 
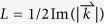
, where 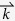 is the complex propagation wavevector and 
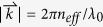
.

### Input power estimation

The average input power from the fiber tip is about 10 mW. The coupling loss between the fiber tip and the waveguide is about 10 dB. However, the exact coupling efficiency may vary with different measurements. In order to keep the input power invariant, we monitor the transmission power at the output port of the waveguide and keep it invariant. Since the optical loss of the waveguide can be neglected, we assume that the input power equals with the output power in numerical simulations.

### Measurements

The central wavelength of the Femtosecond Laser (Del Mar Buccaneer) is 1558 nm, the pulse width is about 300 fs with operating current 560 mA and the repetition rate is 60 MHz. Laser was coupled to the sample by a single mode fiber with a tapered tip (Chuxing Ltd.) and the output power from the tapered tip is about 10 mW. Transmitted wave was coupled to a single mode fiber which is connected with an optical spectrum analyzer (Yokogawa AQ6370).

### Numerical simulations

Short pulse propagation in the waveguide was simulated by a one-dimensional finite-difference time-domain method^27^. The input pulse is assumed to have a Gaussian-like shape in time-domain with width about 300 fs, and has a peak power of about 100 W for the silicon ridge waveguide propagation (Fig. 4b) and a peak power of about 25 W for the graphene/silicon ridge and graphene/silicon slab ridge waveguides propagation (Figures 4b–e). The effective electrical mode areas of the waveguides are about 1 μm^2^. We assume that the light intensity and effective Kerr coefficient distribute homogeneously at this area, and thus can simplify the simulation to one dimension.

### Sample fabrication

Silicon ridge waveguides were fabricated from a 1.5 μm thick SOI wafer. The width of the waveguides is about 1.5 μm and the etched thickness is 0.4 μm. Single layer highly N-doped graphene (XFNano Materials Tech.) was transferred on the surface of the waveguide. Slot waveguides were formed by depositing 40 nm thick SiO_2_ and 200 nm Si_3_N_4_ on top of the graphene to enhance the light density at the graphene layer.

## Additional Information

**How to cite this article**: Liu, K. *et al.* Ultra-fast pulse propagation in nonlinear graphene/silicon ridge waveguide. *Sci. Rep.* **5**, 16734; doi: 10.1038/srep16734 (2015).
